# Advancements in broadband near-infrared spectroscopy instrumentation for the assessment of *in vivo* mitochondrial function: a comparative review and outlook

**DOI:** 10.1117/1.JBO.30.S2.S23914

**Published:** 2025-09-30

**Authors:** Musa Talati, Ilias Tachtsidis

**Affiliations:** University College London, Department of Medical Physics and Biomedical Engineering, London, United Kingdom

**Keywords:** broadband near-infrared spectroscopy, metabolism, spectrometer, cytochrome-*c*-oxidase, hardware

## Abstract

**Significance:**

Broadband near-infrared spectroscopy (bNIRS) provides a noninvasive, nonionizing method for measuring oxidative metabolism through monitoring of cytochrome-c-oxidase oxidation changes. Despite its clinical potential, bNIRS adoption is limited by the complexity, cost, and size of instrumentation. We address the need for a comprehensive overview of bNIRS hardware developments to inform on gaps and propose future device design.

**Aim:**

We catalog and compare bNIRS systems developed over the past 37 years, focusing on hardware configurations, component choices, and trends toward miniaturization and multichannel measurements.

**Approach:**

A systematic literature review was conducted using Web of Science and citation mapping tools. Inclusion criteria targeted systems utilizing broadband light sources and spectrometers, covering a spectral range of 600 to 1000 nm, and capable of measuring CCO concentration changes. A total of 72 articles were reviewed.

**Results:**

The quartz tungsten halogen lamp and commercial bench-top spectrometers remain dominant in bNIRS developments. Recent advancements include fiber-optic innovations and compact charge-coupled device (CCD) based sensors, facilitating multichannel configurations. However, no fully commercial portable bNIRS device exists. The recent addition of micro form factor spectrometers drives the emerging trend toward wearable designs.

**Conclusions:**

Future bNIRS devices must prioritize miniaturization, ease of use, and cost reduction to support clinical translation. Emerging photonic technologies offer promising pathways for compact, wearable bNIRS systems.

## 1 Introduction

The brain is one of the most metabolically active organs in the human body, usually requiring 3.5 mL of $O_{2}$ per 100 g of brain tissue every minute, both in wakefulness and at rest.^1^ It depends on continuous oxygen-rich blood flow to function and meet its metabolic demands; obstructing this results in energy failure and cell death, which causes permanent injury to the brain tissue. There is a global consensus that neuroimaging is vital in the understanding, diagnosis, and prognosis of neurodevelopmental outcomes.^2^^,^^3^

Magnetic resonance spectroscopy (MRS) and positron emission tomography (PET) are two of the most widely recognized methods of noninvasively measuring directly brain metabolism. MRS is considered a benchmark for assessing the effects of cerebral injury,^4^ and this is accomplished with two methods, proton (1H) and phosphorus (31P) MRS. Wilson et al.^5^ presented a review of validated methodologies for the use of 1H MRS in clinical management of brain disease; however, they also outline the lack of widespread adoption due to the technical challenges in obtaining reliable, good-quality results. Liu et al.^6^ provided a similar review of 31P MRS methodologies and suggested that the critical issue limiting clinical application is the balance between long acquisition times or low spatial resolution to achieve adequate signal-to-noise ratios. Both forms also require expensive apparatus to be managed by specialist teams, making their use inflexible and restricted to hospital environments.

PET monitors the uptake of a radioactive glucose tracer, fluorodeoxyglucose (FDG), to inform of energetics within the brain. It has been demonstrated as useful in diagnosing and monitoring cancer and various neurodegenerative conditions.^7^^,^^8^ However, the use of ionizing radiation makes the technique not appropriate for all populations, namely, to monitor neurodevelopment in infants and neonates.

There also exist optical methods for measuring metabolism, such as diffuse correlation spectroscopy (DCS), which utilizes the biological transparency window in the near-infrared (NIR) region of light to measure brain tissue metabolism indirectly through estimations of the cerebral metabolic rate of oxygen (${\mathrm{CMRO}}_{2}$), using blood flow and absolute arterial and tissue oxygenation.^9^^–^^11^

Another optical method was described through the seminal work in 1977 by Franz Jobsis, where it was demonstrated that near-infrared light could penetrate deep into living tissue and thus be used to identify concentration changes of absorbing compounds in said tissues.^12^ Since then, near-infrared spectroscopy (NIRS) has become a recognized tool for research and clinical use, with functional NIRS (fNIRS) being shown to be highly valuable in monitoring functional activity in a wide range of environments,^13^ with sensitive demographics (infants and toddlers),^14^ and at exceedingly low risk to the patient due to noninvasive, nonionizing methods. However, current commercial implementations only provide cerebral oxygenation measurements and have limited ability in measuring the metabolic changes that are important for neurodevelopmental assessments.^15^

These commercial devices often employ only two wavelengths of NIR light for fNIRS measurements, ensuring wavelengths are selected from both sides of the isosbestic point of oxygenated and deoxygenated hemoglobin at ∼808  nm. This is adequate to perform accurate measurements of oxygenation; however, the initial aim of Jobsis’ investigation was to measure *in vivo* changes of oxidation state of cytochrome-c-oxidase (CCO) to monitor metabolic changes. The intrinsic challenge of these measurements arises due to the lower concentration of CCO in cerebral tissue by an order of magnitude when compared with hemoglobin, which dominates the signals.^16^ To combat these effects, systems which utilize multiwavelength (3 to 8 discrete wavelengths) approaches have been developed, with a review in the effectiveness of multiple wavelength combinations by Arifler et al., determining that a combination of eight wavelengths resulted in the lowest mean recovery error of oxCCO concentrations, compared with 3, 4, and 5 wavelength combinations.^17^ This review also identifies a gold standard benchmark for measuring CCO contrast with NIR light, broadband NIRS (bNIRS), which, in this example, utilizes 121 NIR wavelengths (780 to 900 nm) to accurately recover the change in oxCCO concentrations.^18^

bNIRS considers the concept that an increase in the number of utilized wavelengths and the use of spectroscopic algorithms will reduce the effects of chromophore cross-talk and extend this to apply near 100 wavelengths of light through tissue to obtain a robust oxCCO signal while estimating the changes in oxy/deoxy-haemoglobin with a higher accuracy due to increased redundancy and better noise estimation. By utilizing a high number of wavelengths, bNIRS also enables estimations of absolute chromophore concentration and optical path length through second-derivative spectroscopy and broadband fitting of the water and lipid spectra.^19^^–^^21^

This technique, however, leads to an increase in the complexity required of the instrumentation. As such, many bNIRS systems have been developed with varied components and approaches. In this review, we aim to describe and compare the instrumentation of published bNIRS systems over the past 37 years. There exist two reviews, which consider bNIRS systems;^22^^,^^23^ however, the focus of these reviews is the algorithms, protocols, and measurement of the oxCCO signal, via broadband and discrete wavelength instruments. By contrast, the focus of this present review is to document the developments in the hardware and instrumentation of bNIRS systems while highlighting trends and forecasting future technology that may improve cost efficiency, reduce complexity, and decrease form factor.

## 2 Search Method

### 2.1 Defining the Search Criteria

As this review will focus solely on the hardware of bNIRS systems, below, we define the elements required to be considered a broadband system for this review:

1. implementation of a broadband (white light or other continuous NIR spanning) source, which delivers light to the tissue
2. utilization of a detection system that can distinguish the intensity of received light by wavelength
3. collection of an “n” number of wavelengths in a spectrum, not restricted to discrete or specific wavelengths, at every time sample.

The final point is flexible to not exclude any systems where a large range of wavelengths can be collected, but the researchers only recorded a few of them in their study. The definition of broadband NIRS, as presented by Ayaz et al., has been adopted in creating these criteria.^24^ Typical systems following this criterion will record a number of wavelengths in the order of tens or hundreds. It should also be noted that, although not all of these systems might have been used to monitor the redox state of CCO in the brain, these restrictions will ensure that this measurement is possible with any of them.

### 2.2 Search Strategy

**Fig. 1:**
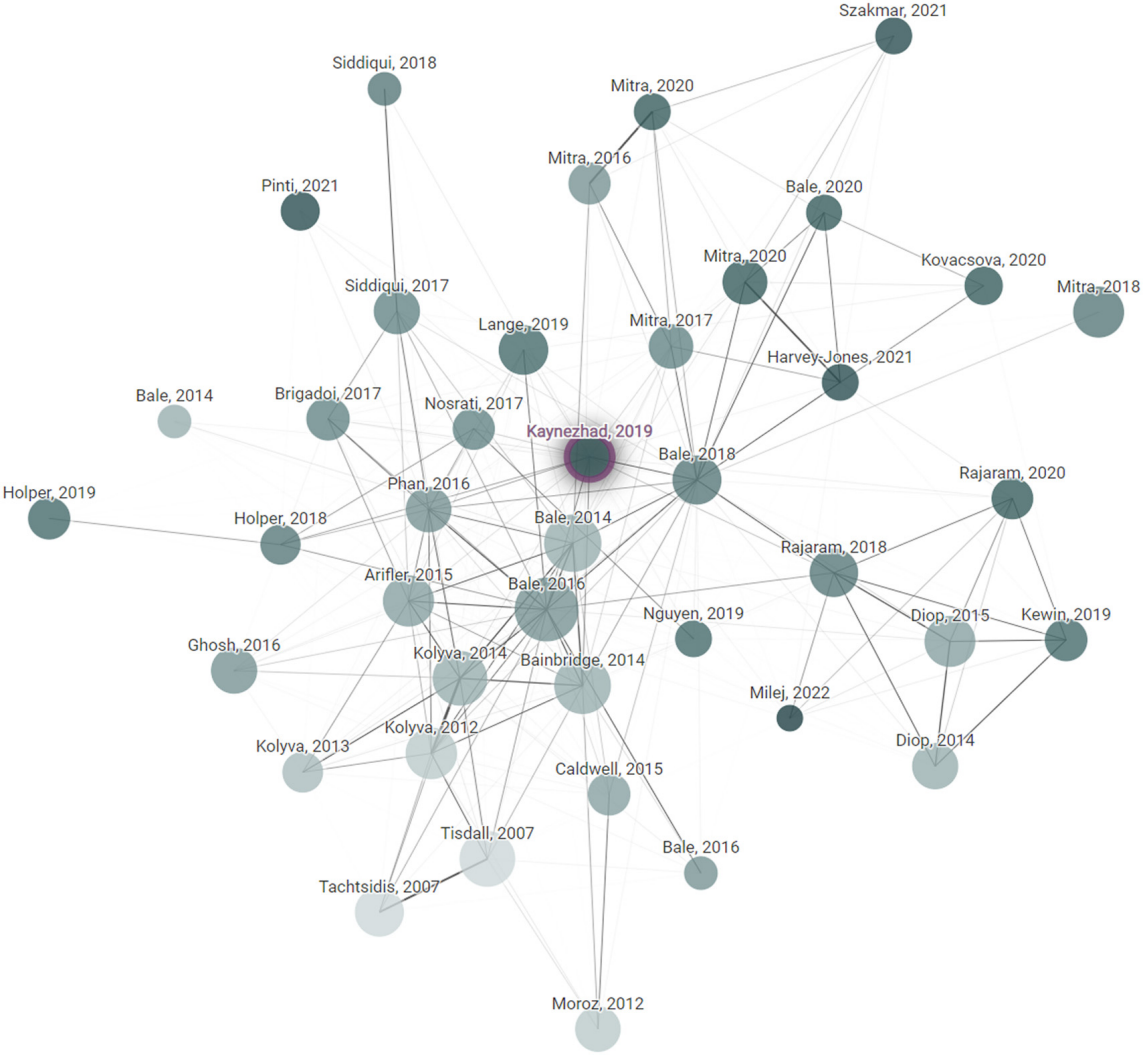
Example of a spider diagram of related articles, by citation and reference, produced at connectedpapers.com.

Papers were identified using the Web of Science and a keyword combination consisting of variations of the terms: near-infrared spectroscopy, NIRS, cytochrome, broadband, bNIRS, and white light. Boolean operators (AND/OR) were used to augment the search to collate papers for the review. The citation mapping tool, Connected Papers, was used to visually identify and locate highly cited, seminal, or foundational papers to add to the database; an example of the citation maps created is shown in Fig. 1. No filtering has been conducted by year because the field of clinical NIRS is relatively new and bNIRS newer still, so this study considers all papers up to 2025.

**Fig. 2:**
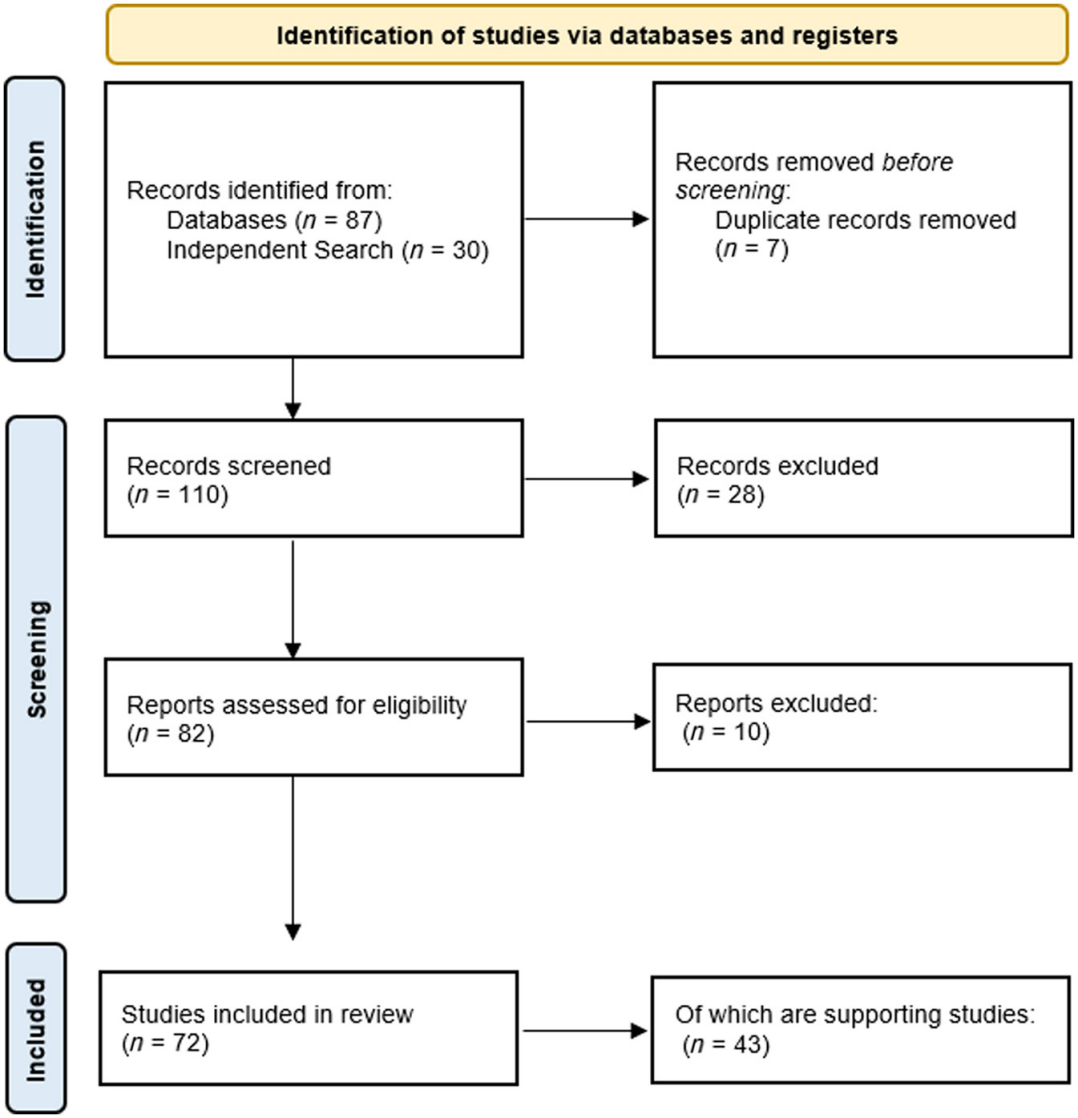
Prisma flowchart of paper selection process.

A PRISMA flowchart has been created to demonstrate the paper selection process (Fig. 2). Publications were included in the review if they presented a novel bNIRS device or supported an existing one with validation studies or subject testing. Abstract-only articles were excluded, and conference proceedings and theses were excluded where a journal article outlining the same system could be found. Studies on animals, humans, and phantoms were included because the main hardware variations between applications on these subjects are in light delivery and not core components (sources and detectors). Articles were also excluded if they contained no information regarding the system hardware.

## 3 Results

The initial search identified 87 articles through databases and 30 through citation tool search, with seven removed as duplicate entries, for a total of 110 articles. After excluding the abstract-only articles, conference proceedings, and nonbroadband NIRS articles, 82 were identified, all of which regarded bNIRS systems. Reviewing the full texts identified that 10 of these articles made no reference to or gave little to no detail regarding the instrumentation employed in the study, leading to an inclusion of 72 articles for the final review. These 72 can be split further into 29 articles where a specific system appears for the first time in publication and 43 articles that gave additional detail on the system or validated its application through measurements. The PRISMA flowchart in Fig. 2 depicts the data collection and selection process.

The results of this review are presented in Table 1; some characteristic details of the systems have been given, including instrumentation models, spectral range and resolution, and number of measurement channels. All systems were used to quantify CCO, either through the primary article or supporting studies, and the initial subject types are given. There has been no recorded use of an entirely commercially available bNIRS device; all studies have constructed their own systems piecemeal with custom-made or “off-the-shelf” components.

**Table 1:** Articles reviewed that presented a novel bNIRS system. Key specifications are displayed along with the instrumentation that comprises the system. Where certain details have not been presented in the work, the cell is left blank.

Primary author	Subject type	Instrumentation	Spectral range	Spectral resolution	Temporal resolution	# of channels
Wray et al.^25^	Rats (head)	250 W quartz halogen lamp, Scanning optical spectrum analyzer (Rofin), GaAs photomultiplier tube (Hamamatsu R636)	700 to 900 nm	∼5 nm	0.2 samples/min	1
Cope et al.^26^	Rats (head)	100 W quartz halogen lamp, custom spectrograph, EEV P8603 CCD	580 to 1004 nm	1.7 nm	1 Hz	1
Nichols et al.^27^	Phantom	250 W quartz halogen lamp, Acton research SP275 spectrograph, Princeton Instruments CCD	510 to 670 nm	0.95 nm	0.25 Hz	15 (within 20 mm)
Matcher et al.^28^	Adult human (forearm, calf, and head)	Tsunami Ti:sapphire laser (Spectra-physics), C1385 Synchroscan streak camera (Hamamatsu)	760 to 900 nm	Sampled every 5 nm		1
Kohl et al.^29^	Adult human (head)	Quartz halogen lamp, SP275 Spectrograph (Acton Research), CCD detector (Princeton Instruments)	700 to 1000 nm	20 nm	50 Hz	1
Bevilacqua et al.^20^	Phantom	150 W quartz halogen lamp (Fibre-Lite), S2000 Spectrograph (Ocean Optics), Linear CCD detector	650 to 1000 nm	5 nm	0.1 Hz	1
Klassen et al.^30^	Adult human (head)	50 W quartz halogen lamp, holographic spectrometer (ScienceTech),	Peltier-cooled CCD	600 to 1000 nm	4 nm	10 Hz
Brown et al.^31^	Piglet (head)	Quartz halogen lamp, custom holographic spectrometer, CCD camera (Wright Instruments)	600 to 980 nm	0.40 nm		1
Xu et al.^32^	Phantom, rats (head)	100 W quartz halogen lamp (Oriel Triax 320 m Spectrograph (JY Horiba), Instruments, 77501), CCD EEV CCD05-30-219 (Wright Instruments)	700 to 900 nm	0.33 nm		64
Geraskin et al.^33^	Adult human (forearm)	(1) 50 W quartz halogen lamp, SP-150 spectrometer (Acton Research), Spec-10 CCD (Roper Scientific), (2) LXHL-LW5C White LED (Luxeon), USB2000 spectrometer (Ocean Optics)	(1) 550 to 1000 nm	(1) 3.4 nm	(1) 0.4 Hz	(1) 6
			(2) 450 to 700 nm			(2) 1
Tisdall et al.^34^	Adult human (head)	Quartz halogen lamp, 270 M spectrograph (Instruments SA), CCD (Wright Instruments)	650 to 980 nm	∼5 nm	1 Hz	1
Diop et al.^35^	Piglet (head)	50 W quartz halogen lamp, holographic spectrometer (ScienceTech Inc.), S7010-1007 CCD (Hamamatsu)	600 to 1000 nm	0.38 nm	5 Hz	6
Lee et al.^36^	Rabbit	HL-2000 halogen source (Ocean Optics), MS127i Spectrometer (Oriel), InstraSpec IV CCD	600 to 1000 nm			1
Pucci et al.^37^	Phantom	Avalight-HAL halogen lamp, QE65000 Spectrometer (Ocean Optics)	650 to 1100 nm		0.2 Hz	1
Tachtsidis et al.^38^	Phantom, Adult human (forearm)	50 W quartz halogen lamp, lens imaging spectrometer, PIXIS 512f CCD (Princeton Instruments)	504 to 1068 nm	4 nm	0.25 to 1 Hz	4
Wright et al.^39^	Piglet (head)	HL-2000-HP halogen lamp (Ocean Optics), QE65000 spectrometer with modified entry (Ocean Optics)	4.7 nm	2.5 Hz	1	
Bale et al.^40^	Newborn human (head)	100W quartz halogen lamp (Oriel Instruments, 77501), Acton LS785 (Princeton Instruments), PIXIS 512f CCD (Princeton Instruments)	770 to 906 nm	0.7 nm	1 Hz	8
Phan et al.^41^	Adult human (head)	2× 50W quartz halogen lamps, custom lens spectrographs, PIXIS 512f CCDs (Princeton Instruments)	780 to 900 nm	∼1.1 nm	0.35 Hz	24
Nosrati et al.^42^	Adult human (head)	OSL2BIR halogen lamp (Thorlabs), 2× QE65000 spectrometers (Ocean optics)	700 to 900 nm	0.4 nm	1 Hz	2
Wang et al.^43^	Adult human (forearm)	Model 3900 halogen lamp (Illumination Tech.)	(1) 450 to 1100 nm			1
		(1) i-trometer (B&W Tek)	(2) 735 to 1100 nm			
		(2) QE-Pro (Ocean Optics)				
Kaynezhad et al.^44^	Piglets (head)	HL-2000-HP halogen lamp (Ocean Optics), Ventana VIS-NIR (Ocean Optics)	430 to 1100 nm	4 nm	0.1 Hz	1
Thiele et al.^45^	Rat (head)	LH-150 halogen lamp (Pentax), 3× USB2000 VIS-NIR spectrometer (Ocean Optics)	600 to 900 nm	1 nm		2
Rajaram et al.^46^	Newborn human (head)	HL200HP 10w halogen lamp (Ocean Optics), Custom spectrometer, iDus Andor camera	548 to 1085 nm	1.65 nm	4 Hz	2
Dutta et al.^47^	Cerebral organoid	SL1 tungsten halogen source (StellarNet), Silver-nova spectrometer (StellarNet)	490 to 900 nm			1
Hashem et al.^48^	Mouse (head)	100 W quartz halogen lamp (Oriel Instruments, 77501), Shamrock 303i spectrograph (Andor Tech), DU420-BR-DD CCD (Andor Tech)	700 to 967 nm	3.7 nm	6 Hz	1
Wang et al.^49^	Adult human (head)	Model 3900e light source (Illumination Tech), Teledyne Princeton Instruments Spectrograph	780 to 900 nm		4 Hz	2
Vezyroglou et al.^50^	Newborn human (head)	2× HL-2000 halogen lamps (Ocean optics), Acton LS785 (Princeton Instruments), PIXIS 1300F CCD (Princeton Instruments)	610 to 918 nm	1 nm	2 Hz	16
Oh et al.^51^	Rat (head)	HL-2000-HP halogen lamps (Ocean Optics), USB4000 16-bit CCD Spectrometer (Ocean Optics)	Unspecified, 780 to 900 nm used in the algorithm	1.5 nm	1 Hz	1
Talati et al.^52^	Adult human (forearm)	SMBBIR45A broadband LED (Ushio), C14384MA SMD-type spectrometer (Hamamatsu)	770 to 906 nm	14 nm	1 Hz	2

**Fig. 3:**
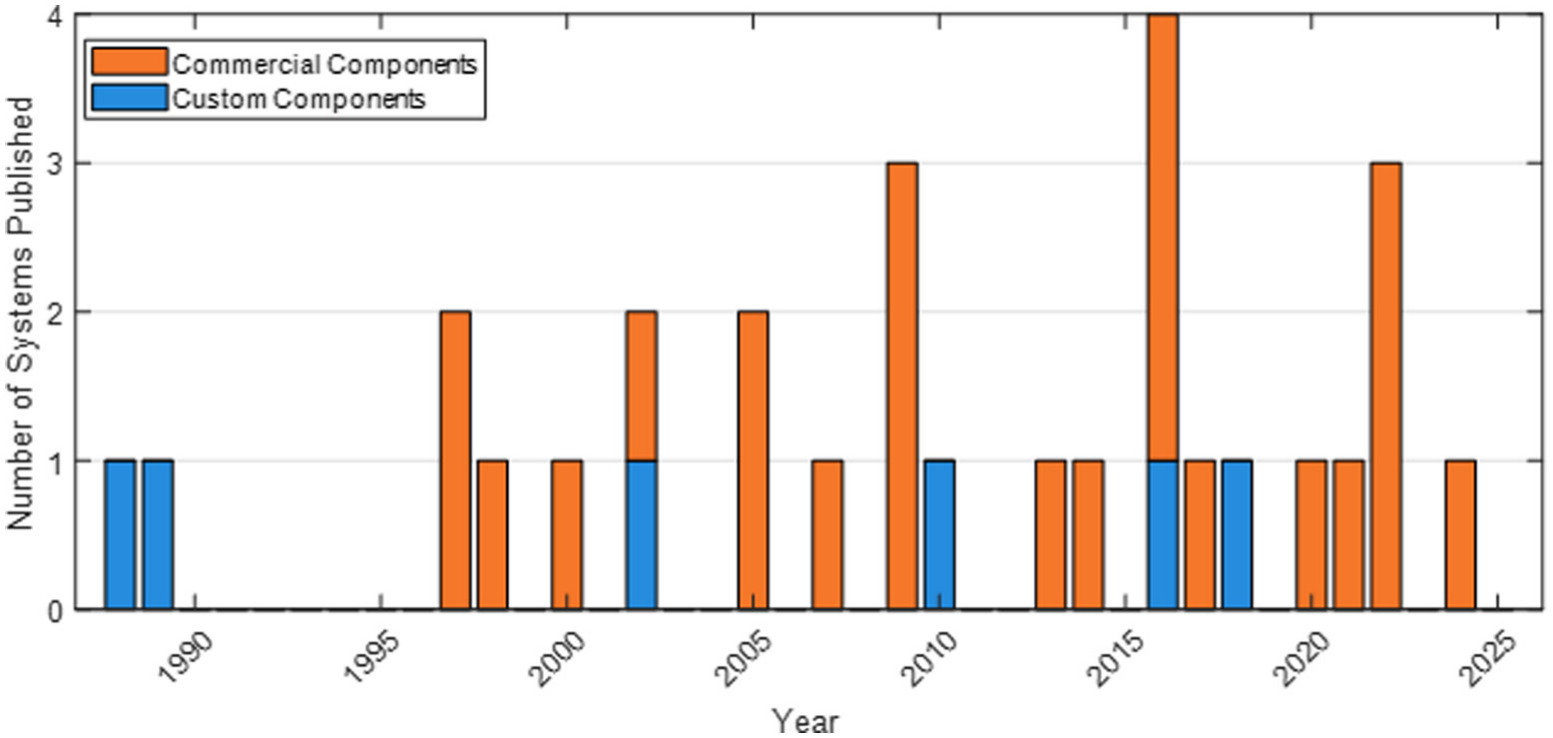
Chart showing the number of novel bNIRS systems developed each year from 1988 to 2025. Blue bars show the devices created with any amount of custom components; orange bars are for when only commercially available components were used.

From Fig. 3, it is apparent that there is a growing number of bNIRS systems being developed. With a consistent rate of new systems being reported over the last 25 years and spikes in the years 2005, 2009, and 2016, between 1989 and 1997, there appears to be a drop in interest for new systems; however, a review of the papers published between those times asserts that the field was still active. It is clear that custom components were necessary to create the first systems, so it is possible that newer systems were only created when commercial devices that could be utilized in bNIRS became more readily available. In the past 3 years, since 2022, there has been only one publication in bNIRS system development, by Talati et al.;^52^ however, there have been many articles that utilize the systems cataloged previously, supporting the idea that bNIRS instruments are still well maintained.

Trends in spectral range and spectral resolution can give some insight into the effectiveness of the systems over time. Combining the two specifications gives an idea of the total number of wavelength spectra each device could record, which informs on the precision of the CCO measurements.^18^ There does not appear to be any appreciable trends in the evolution of spectral range in the time frame, with the early system bandwidths starting around 400 nm, and in the most recent wave, most systems have largely varying bandwidths. Spectral resolution is very consistently below 5 nm for all systems, except one, regardless of year published.

**Fig. 4:**
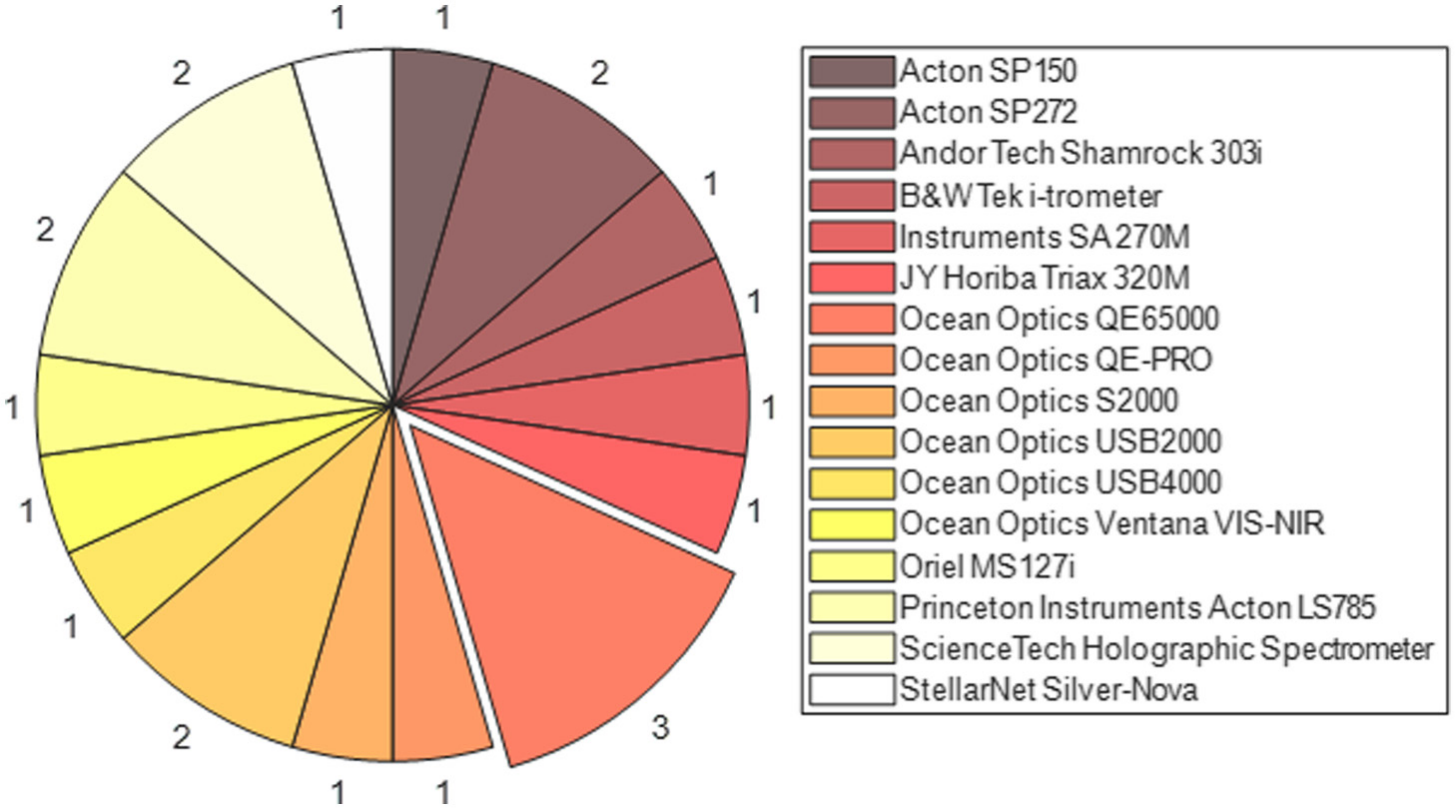
Proportion of devices that use the same commercially available spectrometer. With Ocean Optics QE65000, the most frequently used, at three devices.

**Fig. 5:**
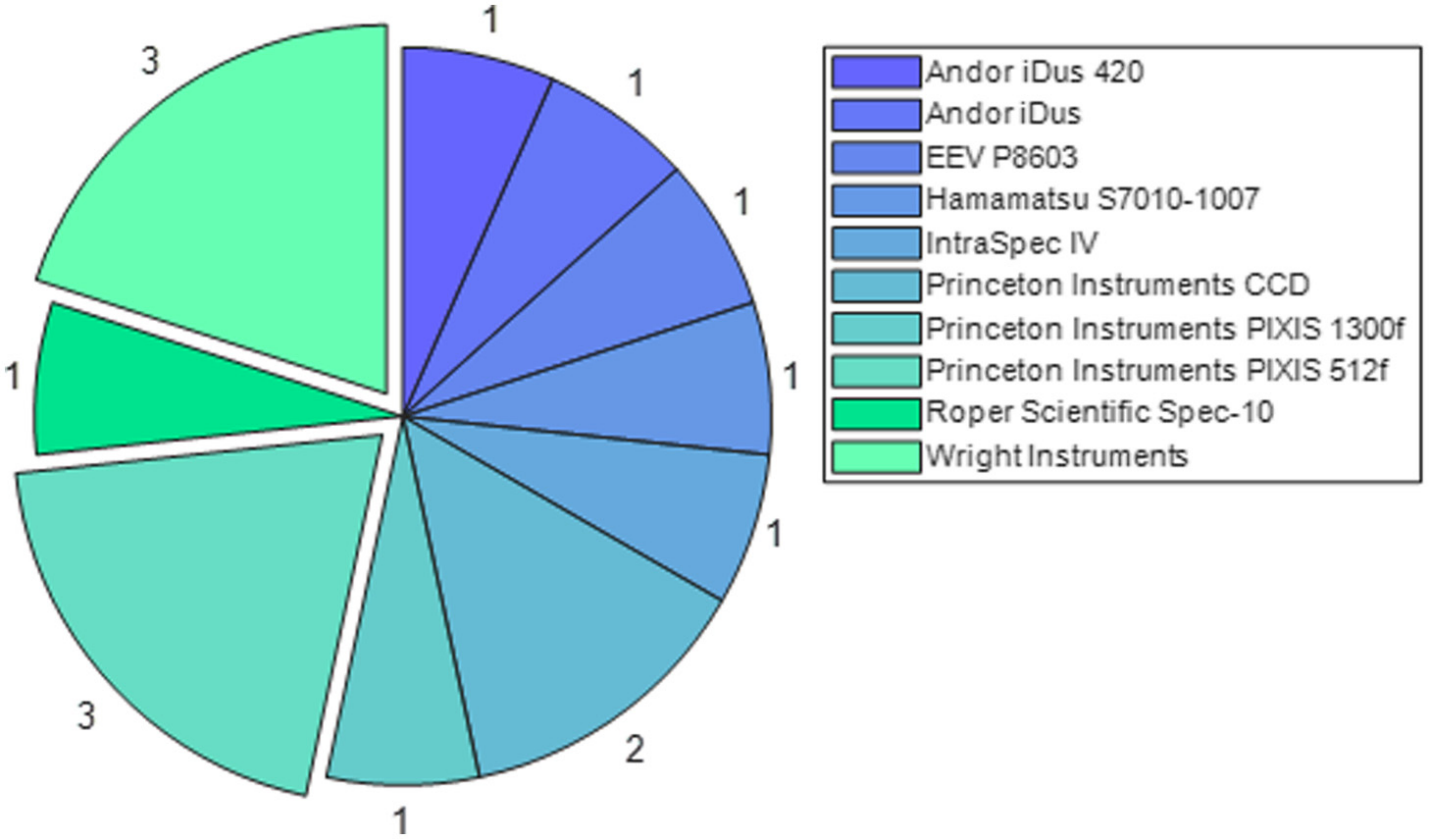
Proportion of devices that use the same commercially available CCD. With Wright Instruments CCD and Princeton Instruments PIXIS 512f being the most frequently used, at three devices each.

For the systems that make use of commercially available devices, the popularity of each component is shown in Figs. 4 and 5 for spectrometers/spectrographs and CCDs, respectively. The majority of the systems use commercially available CCDs and spectrometers or spectrographs, with the most popular spectrometer being the Ocean Optics QE65000 and the most popular sensor being the Princeton Instruments PIXIS 512f.

Although this is a comprehensive review of the bNIRS systems published in the past 35 years, some system specifications are not entirely reported in every article and will have to be omitted from the specification table.

### 3.1 System Overview

**Fig. 6:**
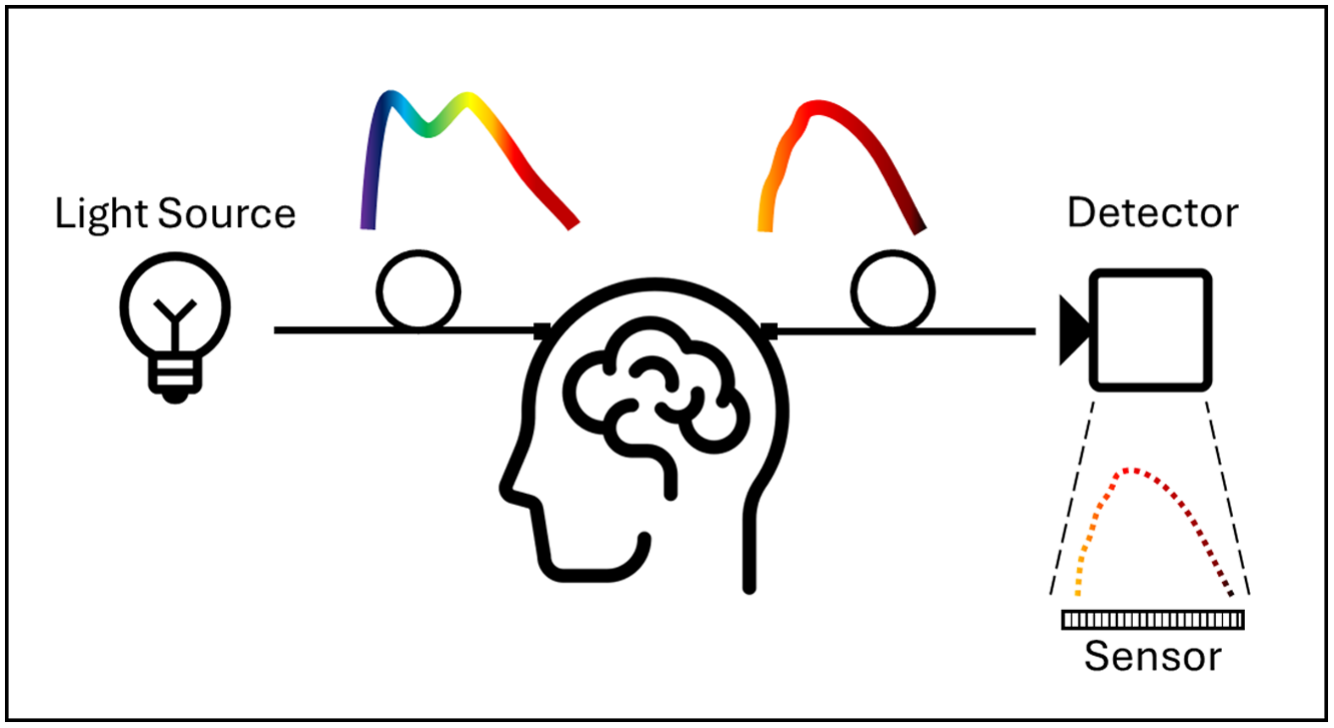
Schematic of the system design typically used in bNIRS. The light source provides continuous or white light, which is delivered to the subject. At the detector, light distinguished by wavelength is collected on the sensor pixels. The subject of the system is not limited to the human head.

Typical bNIRS systems consist of a source, a detector, and a form of light delivery. The detector consists of a device to separate intensities by wavelength and a sensor to collect the intensity values electronically for chromophore calculation. Figure 6 shows the basic outline of these systems. All systems in this review follow this example.

#### 3.1.1 Sources

In all but three systems reported, a form of quartz tungsten halogen lamp (QTH) was used for illumination of the subject. The QTH provides a very smooth high-intensity spectrum across the visible and NIR regions, and the glass is a filter for UV light, making it the most popular choice for bNIRS systems. Alternative high-pressure arc lamps such as Xenon and Mercury lamps are less useful for bNIRS investigations due to the number of peaks in their NIR spectra, which is a trait that makes them popular for fluorescence excitation instead. QTH are also extraordinarily stable, when run in DC at nominal wattages typical short-term stability levels are <0.02%/h.

Many different models of QTH have been used but with the typical set-up consisting of an aspheric lens, or other collimating and focusing tool, used to couple as much light into an optical fiber as possible. A reference fiber is used at times to ensure that any variations in intensity of the source are recorded for accurate attenuation change calculations (Nichols et al.^27^), or an integrating sphere is employed with one source fiber and one detection fiber to record the source spectrum (Bevilacqua et al., Geraskin et al.).^20^^,^^33^

The spectral range of a QTH can span from around 400 nm to almost 2500 nm. As the biological window runs from 650 to 1350 nm, this is a clear overshoot of the required wavelengths.^53^ Filters are implemented at the source to reduce the excess light before it is transferred to tissue. Long pass filters allow light above a designated wavelength to pass through, typically 650 nm, eliminating shorter wavelengths, reducing total power output, and reducing possible UV exposure (Wray et al., Xu et al., Tisdall et al., Kaynezhad et al., Rajaram et al., Vezyroglou et al.).^25^^,^^32^^,^^34^^,^^44^^,^^46^ Similarly, to reduce heating effects of the long IR range, short pass filters can be employed at the 1000 nm mark to remove those longer wavelengths (Cope et al., Tachtsidis et al.);^26^^,^^38^ these are also available in the form of a water filter (Wray et al.).^25^ Combinations of both long and short pass filtering, or bandpass filtering, combats both potential issues (Klassen et al., Bale et al., Phan et al.).^30^^,^^40^^,^^41^

As the QTH sources are designed to run continuously, requiring a warm-up time to reach maximum intensity and stability, shutting off a source for channel multiplexing is not feasible. For this purpose, shutters are used, with an adjustable iris to modulate the intensity of the source (Diop et al., Hashem et al.), ^35^^,^^48^ or solenoid electronics to cut off the light at precise times (Xu et al., Bale et al., Phan et al., Wang et al., Rajaram et al.).^32^^,^^40^^,^^41^^,^^43^^,^^46^

The first of the systems that does not use a QTH was produced by Matcher et al. in 1997.^28^ The system was developed to measure the wavelength dependence of tissue-scattering coefficients in the NIR range and used a pulsed mode-locked Ar pumped Ti:sapphire laser. This was a time-resolved spectroscopy system, so the information was resolved from the tissue temporal point-spread function. To cover a broadband range, the wavelength output of the laser was tuned to cover 760 to 900 nm in 5 nm steps. This is not a continuous spectrum acquisition at all time points, unlike the other bNIRS systems; however, it does span the NIR range with many wavelengths and has been included in the review due to its relevance as one of the earliest broadband spectrum instruments.

The second system of note is continuous and uses a white light source also; however, it is an LED instead of a QTH. The secondary system by Geraskin et al. reported the use of a white light LED (Luxeon LXHL-LW5C),^33^ which has a spectral range of 450 to 700 nm and was coupled to an optical fiber. This device was used to measure the skin oxygenation by visible reflectance spectroscopy alongside the main bNIRS system, so the LED is not directly tied to the bNIRS monitoring.

Finally, the system by Talati et al. uses a broadband phosphor-converted LED to provide NIR light in the range 400 to 1000 nm.^52^ Unlike the previous LED system, this device forgoes optical fibers, instead placing the LED dome directly onto the subject. This setup was used to directly probe changes in oxygenation and metabolism through bNIRS and can demonstrate the potential for direct LED integration into future studies

#### 3.1.2 Light delivery

For light transport from the source to the subject and then collection from the subject to the detectors, the most popular choice is the optical fiber. Fiber optics can be thought of as “light pipes,” guiding light through twists and turns to the desired location via total internal reflection. The numerical aperture (NA) of an optical fiber is the dimensionless measure of the largest angle an incident ray can have for total internal reflection. To increase accuracy at the detector while maintaining good SNR, NA is considered throughout the light delivery process to reduce the error from stray light. Systems employ high NA fibers to collect and deliver as much light as possible (Cope et al., Diop et al., Bale et al., Vezyroglou et al.).^26^^,^^35^^,^^40^^,^^50^ Light throughput can also be increased using silicone oil as a coupling gel to maintain good optical contact (Wray et al.).^25^

The fibers that terminated at the subject will have a bare end to collect or deliver light to the medium. Hashem et al. affixed a gradient-index lens to the end of the fiber, focusing the light into the subject and collimating the received light into the fiber.^48^ A plastic shroud covering a 90-deg bend in the fiber, forming a flat head that can be affixed to the head of a subject, minimizing pressure caused to the skin (Bale et al., Wang et al., Vezyroglou et al.).^40^^,^^43^^,^^50^

Single-core fibers are implemented in a linear fiber array by Nichols et al.,^27^ taking each 200  μm fiber from the probe to a 400  μm fiber that runs to the detector, in a 1:1 alignment so that they can pass through a gradient neutral density (ND) filter. The ND filter is used to reduce the intensity of the signal from the detection fibers that are positioned close to the source fibers, reducing the crosstalk effects of the strongest channel at the detectors. Xu et al. employed an ND filter while also giving the weaker channels greater pixel height on the CCD and the stronger channels less.^32^

One method of increasing the signal-to-noise ratio is using fiber optic bundles. A fiber optic bundle is any fiber assembly that contains more than one fiber optic in a single cable. These fiber bundles can also be bifurcated, split into two probes, to produce two channels from a single source (Phan et al.),^41^ or reorganized within the cable from “round to slit.” The round to slit fiber bundle works by taking the multiple fibers from a light-gathering round configuration to a stacked strip of fiber outputs, which are closer in shape to the slit of the spectrometer, decreasing the coupling losses from light blocked by the entry slit (Cope et al., Nichols et al., Tachtsidis et al.).^26^^,^^27^^,^^38^ This also allows multichannel systems to record the channels simultaneously on one CCD using the slit’s vertical axis to separate each channel (Xu et al., Bale et al., Phan et al., Vezyroglou et al.).^32^^,^^40^^,^^41^^,^^50^ Three systems move a step further and increase light throughput by removing the entrance slit to the spectrometers altogether and instead using the slit end of the fiber as the spectrometer entrance (Diop et al., Nosrati et al., Kaynezhad et al.).^35^^,^^42^^,^^44^

#### 3.1.3 Detectors

As shown in Table 1, the typical instrument for optical detection and measurement by wavelength is a spectrometer. The only systems that do not use some form of the spectrometer are the system by Wray et al.,^25^ which uses an optical spectrum analyzer to characterize the absorption spectra of cytochrome, and the system by Matcher et al.,^28^ which uses a streak camera to measure tissue scattering coefficients. The optical spectrum analyzer is used to sample the received light between 700 and 1000 nm in steps of 5 nm, leading to 60 spectra taken but not simultaneously, similar to Matcher’s nonsimultaneous laser-based system, which samples the NIR region in steps.

As the subjects used in each study vary and not all details are reported, it is difficult to generate a consistent metric for each system. There exist several protocols that can generate a measure of effectiveness for NIRS systems; the MEDPHOT protocol evaluates the capabilities of instruments to measure absorption and reduced-scattering coefficients; the basic instrument protocol (BIP) measures instrumental characteristics, and the nEUROPt protocol assesses the performance of TD systems in detection, localization, and quantification of absorption changes in the brain.^54^ The nEUROPt protocol has been adapted to use in CW systems and therefore can be implemented across bNIRS; however, this is not a common practice across the studies in this report.

A possible metric that can be considered is the number of wavelengths the system can reliably measure, which is highly dependent on the spectrograph choice. From the system’s information available, this can be calculated by determining the spectral range of the system and dividing it by the spectral resolution. However, this does not account for the pixel binning (summing of multiple pixel intensities at the CCD) that may be practiced to increase SNR. As such, these spectrometer specifications are given where available in Table 1 but will not be used explicitly to compare each system.

Tachtsidis et al., Bale et al., Phan et al., and Vezyroglou et al. utilize a lens-based spectrograph,^38^^,^^40^^,^^41^^,^^50^ instead of the traditional mirror-based spectrographs that the other spectrometer-driven studies use. These lens-based spectrographs are reported as providing a higher efficiency than mirror-based ones with regard to light throughput (over 99% transmission throughout the entire working range of the spectrograph).^40^ However, the Czerny–Turner configuration of the mirror spectrographs can result in a smaller device.

In this report, the spectrographs are differentiated further by the type of grating used to diffract the light into its constituent wavelengths. Ruled gratings can typically achieve higher efficiencies than holographic gratings due to their blaze angles, and these are the popular choice in spectrometers used by bNIRS systems. However, the holographic grating can increase the SNR of the signal by minimizing the stray light and was the choice for five of the systems (Klassen et al., Brown et al., Diop et al., Wang et al., Kaynezhad et al.).^30^^,^^31^^,^^35^^,^^43^^,^^44^

Many specifications of the spectrometer depend on slit width, including spectral range, resolution, and throughput. The device first reported by Kohl et al. is an outlier among bNIRS instruments as it has a resolution of 20 nm,^29^ whereas the others have resolutions of 5 nm or less. Later, the same device was used by Kohl et al., and the slit width was reduced from the 1 mm reported previously, giving an effective resolution of 10 nm.

Nearly all detectors used functioned as stationary, detached systems that required optical fibers to connect to the subject. Most systems are not at a point that could be considered portable, but innovations in light delivery and improved SNR mean that spectrometers that are becoming smaller each year can be used with greater efficacy. The Acton Research SpectraPro 275, used by Nichols et al. and Kohl et al., has the dimensions 388×210×178  mm and weighs 9 kg;^27^^,^^29^ compare this to the Ocean Optics QE65000, used by Pucci et al., Wright et al., and Nosrati et al.,^37^^,^^39^^,^^42^ which has dimensions 182×110×47  mm and weighs 1.18 kg, which is 15× smaller in volume and significantly lighter. In the most recent study, the Hamamatsu C14384MA is used by Talati et al.^52^ This is an SMD-type spectrometer with dimensions 11.5×4.0×3.1  mm and weight of 0.3 g, now 5 orders of magnitude smaller than the most popular mini-spectrometer and integratable into a wearable system.

#### 3.1.4 Sensors

Wray et al. begin recording whole-spectrum measurements with a photomultiplier tube (PMT) for a sensor.^25^ The measurements made with this system are not continuous across the whole spectrum, as the spectrum analyzer scans through each wavelength, an intensity reading can be output from a nonwavelength discriminating device and recorded. The PMT takes low photon count signals and cascades them into greater currents than a photodiode, making it a very sensitive device, but limited to this application.

Since then, the silicon-based CCD has become a popular choice because of its high quantum yield, low dark counts, and capabilities in multichannel measurements. The CCD can be constructed in a linear array or 2D design. Bevilacqua et al.^20^ used the linear CCD integrated with the Ocean Optics S2000 spectrometer with dark noise of 3.5 RMS counts. Thiele et al.^45^ used the linear CCD integrated with the Ocean Optics USB2000 with dark noise of 2.5 RMS counts. Neither of these CCDs is reported to have cooling.

The 2D CCDs allow for an axis to be wholly or partially used for binning, reducing the resolution while improving SNR. Nichols et al. conducted binning of 10 pixels along the slit axis and 3 pixels along the wavelength axis, creating a “superpixel” and limiting the resolution to 0.95 nm.^27^ Similarly, Geraskin et al. binned 10 pixels over the wavelength axis and the entire vertical axis,^33^ whereas Klassen et al. only binned vertically across 128 pixels, setting the resolution to 0.38 nm/pixel.^30^

As mentioned in the light delivery section, the modified fiber bundles combined with a 2D CCD allowed for the vertical axis to be separated into each channel to be recorded simultaneously (Xu et al., Bale et al., Phan et al., Vezyroglou et al.).^32^^,^^40^^,^^41^^,^^50^

Two popular CCD devices were the integrated CCD of the Ocean Optics QE65000 (Pucci et al., Wright et al., Nosrati et al.)^37^^,^^39^^,^^42^ and the Princeton Instruments PIXIS:512f (Tachtsidis et al., Bale et al., Phan et al.).^38^^,^^40^^,^^41^ The QE65000 uses a 2D back-thinned CCD with thermoelectric cooling to −15°C and a dark current of 200 e per pixel per second at 0°C. The PIXIS 512f is a 2D front-illuminated CCD with thermoelectric cooling to −70°C and a dark current of 0.002 e per pixel per second.

Other 2D front-illuminated CCDs are the S7010-1007 by Hamamatsu Photonics; using a four-stage Peltier cooling system to reduce the temperature to 200 K, with a dark current of 0.0077 e per pixel per second (Diop et al.),^35^ and the PIXIS 1300f; with cooling to −70°C and a dark current at −60°C of 0.01 e per pixel per second.

Examples of integrated back-thinned CCDs in detection systems are the B&W Tek i-trometer (Wang et al.)^43^ and the Ventana Vis-NIR (Kaynezhad et al.).^44^ The iDus Andor camera provides thermoelectric cooling to −100°C to reduce dark current in its back-thinned CCD (Rajaram et al.),^46^ and the Andor Technology DU420-BR-DD back-thinned 2D CCD is cooled to −40°C the same way (Hashem et al.).^48^

Thermoelectric cooling is the primary choice of cooling for CCDs, and the previous LN2 systems would require extra space and apparatus to adequately cool, as well as the need to replace the nitrogen coolant when expended (Cope et al., Nichols et al.).^26^^,^^27^

#### 3.1.5 Channels

We consider a channel to be the combination of a source-detector pair some fixed distance apart, and we expect each source-detector pair to sample different tissue volumes. The majority of studies consisted of one-channel systems, with some distinct outliers.

Two early systems had many channels, 15 and 64, that were all densely packed onto a probe holder separating each source and detector probe by only 1 to 2 cm (Nichols et al., Xu et al.).^27^^,^^32^ In a muscle oxygenation study, six detection probes were arranged on the skin in a line, separated by 2.5 mm each, with a light probe affixed at a mean distance of 35 mm away, resulting in a six-channel system that monitors the muscle at depth (Geraskin et al.).^33^ A shutter system can be employed to multiplex the detection fibers, ensuring only the channel with an open detection optode at the end is resolved at the spectrometer (Diop et al.).^35^

The multichannel system proposed by Tachtsidis et al. uses shutters to ensure no cross-talk between the FD system and the bNIRS one,^38^ but as there is only one source, the four channels created by the detectors at differing source detector separations (SDSs) can be projected across the vertical axis of the CCD. Bale et al. used the same principle, extended across two sets of sources and detectors,^40^ with eight channels across the vertical axis. Phan et al. went one step further for the extensive 24 channels using time multiplexing for two sources that end in bifurcated fibers and two independent spectrographs.^41^ With the developments in sensor pixel density, a larger CCD was used, similar to the eight-channel system, to create a 16-channel system with a single spectrometer and 16 fiber stack slit (Vezyroglou et al.).^50^

When creating multichannel systems with linear CCDs, the option of using the vertical axis of the CCD chip for channel separation is no longer possible, and so, multiple spectrometers can be used with their own detection fibers (Thiele et al.).^45^

**Fig. 7:**
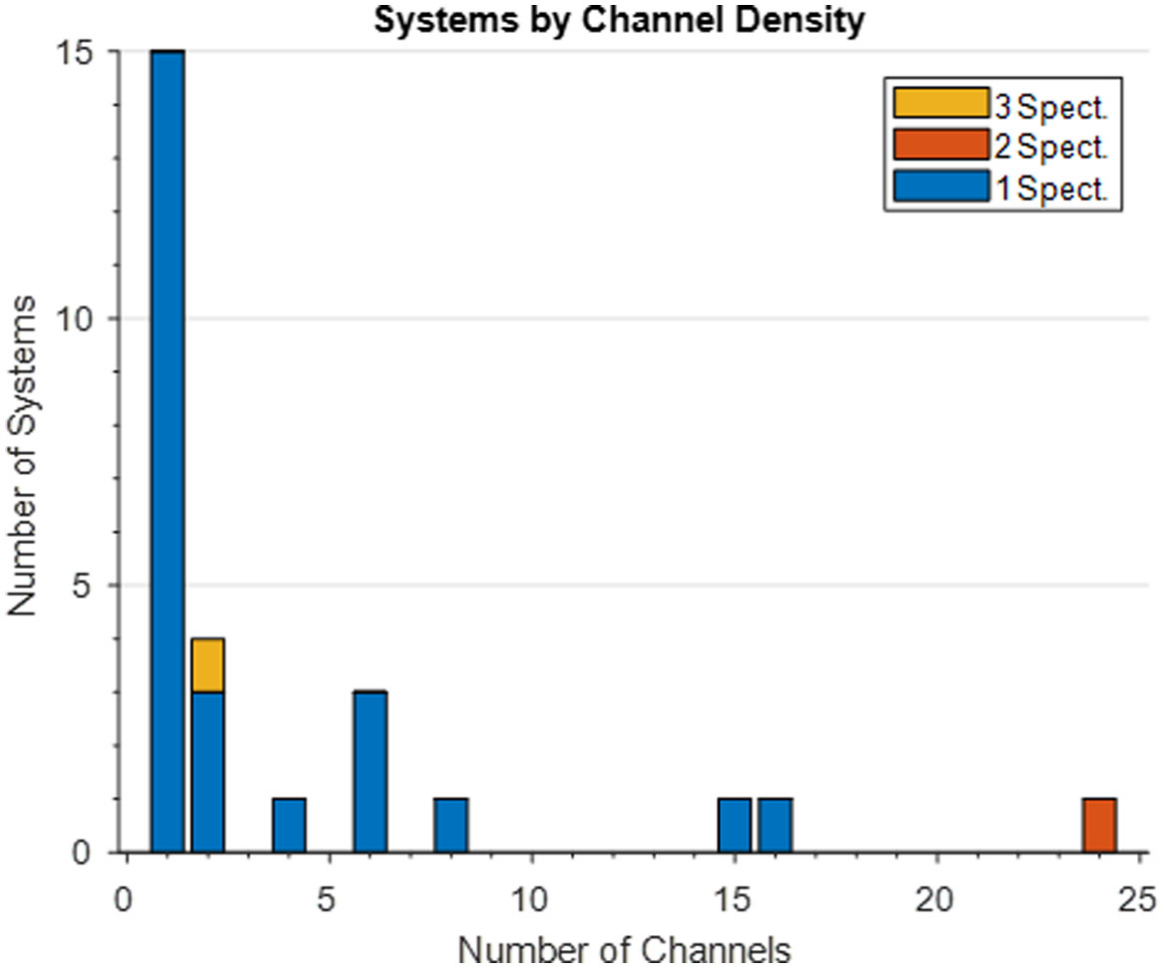
Stacked bar chart visualizing the channel density of systems in this review. The bar size indicates the number of systems, with the color denoting how many spectrometers those systems utilized, blue for one, orange for two, and yellow for three. One system with 64 channels and one spectrometer has been excluded from this plot for clarity.

A summary of the channel density of systems highlighted in this section can be seen in the Fig. 7. Channel density here refers to the number of channels produced per spectrometer, not the spacing between channels.

#### 3.1.6 Hybrid instruments

To validate, calibrate, or provide additional insights to the broadband studies, often simultaneous tests are performed with other functional imaging modalities.

Frequency domain (FD) systems are integrated to determine the coefficients of absorption and scattering in the medium and measure absolute attenuation across the NIR wavelength range with the broadband systems (Bevilacqua et al., Lee et al., Tachtsidis et al.).^20^^,^^36^^,^^38^ The use of pulse oximeters can be used to find pulse rate and arterial saturation, which can in turn be used to determine the wavelength dependence of the pathlength in the human head (Klassen et al.)^30^ or the change in cerebral concentration of CCO during hypoxia (Tisdall et al.).^34^

Xu et al.^32^ reported a novel magnetic resonance (MR)-coupled broadband NIR tomography system, which uses MR imaging (MRI) to validate changes in blood-oxygen levels by the bNIRS system and provides images of structures for the external and internal tissue boundaries to improve spatial resolution of the properties in each tissue type. MR is also used by Bale et al. to statistically validate the system for use in measuring change in blood oxygenation and CCO.^40^

For validation of the cerebral blood flow (CBF) made by the broadband system in the article by Diop et al.,^35^ a computed tomography (CT) perfusion was conducted. Photobiomodulation (PBM), low-level laser therapy, was conducted, and the change in CCO and hemoglobin concentrations was measured, noting a significant increase in oxidized CCO and ${\mathrm{HbO}}_{2}$ concentrations at the site of laser energy on the forearms of adults (Wang, Tian et al., Wang, Ma et al.),^43^^,^^49^ and on rats’ heads (Oh et al.).^51^

DCS is a noninvasive technique that measures cortical blood flow by laser illumination, Rajaram et al. combined this with bNIRS to enable continuous monitoring of absolute cerebral blood flow and changes in oxidized CCO concentrations. In the broadband NIRS monitoring of an organoid generated from a schizophrenic cell line, electrophysiological investigations were run using a tetrode array (Dutta et al.).^47^

Video-electroencephalogram (EEG) measurements were made across 12 channels simultaneously with bNIRS monitoring in a child with extensive malformation of cortical development in the left posterior quadrant, causing seizures. The combined system is reported to have given unique insights into the location of the epileptic focus and the changes in brain physiology (Vezyroglou et al.).^50^

## 4 Discussion

Broadband near-infrared spectroscopy is a powerful tool in assessing the cerebral metabolism and oxygenation and is considered the gold standard for measuring the concentration changes of oxidized CCO *in vivo*. However, the implementation of bNIRS requires specialized and specific instrumentation, including broadband light sources, spectrometers, and high-efficiency detectors, which leads to an increase in system complexity, cost, and size.

Our review of bNIRS instrumentation shows that the majority of systems employ commercially available CCDs and spectrometers, with the Ocean Optics QE65000, a device developed in 2001, being the most commonly used spectrometer, but only as it appears in three distinct systems. Generally, where single-channel systems are concerned, the choice of spectrometer is varied between a few NIR capable options. Of all systems reviewed, 90% use a QTH lamp, of varying power and construction, as a broadband source, due to its stable spectral output in the NIR range. Although alternative sources such as LEDs and supercontinuum lasers have been explored, the QTH remains the predominant choice due to its well-documented usage and widespread availability.

A key trend in bNIRS development is the transition from a single channel to multichannel systems. Although single-channel designs still constitute the majority, with more than 50% of systems falling into this category, there has been a clear shift toward multichannel configurations that enhance spatial resolution and enable regional brain monitoring. The integration of fiber bundles and advanced CCDs has facilitated the development of a system capable of simultaneous multisite measurements, addressing the need for more comprehensive cerebral monitoring. This has not yet been extended to miniature spectrometer implementations (with linear detectors), except when using multiple spectrometers.^55^

As bNIRS moves toward clinical applications, there is a growing interest in its ability to directly monitor mitochondrial function. Several studies, including those reviewed here, have demonstrated the potential of bNIRS for assessing metabolic dysfunction in conditions such as neonatal brain injury, hypoxia, and neurodegenerative diseases, through the understanding of mitochondrial function.^22^^,^^56^^–^^58^ However, for widespread clinical adoption, future bNIRS instruments must prioritize miniaturization, ease of use, and wearability. Advances in compact spectrometers, fibreless probes, and wireless technology will be crucial in making bNIRS systems more accessible for bedside and mobile monitoring.

### 4.1 Multiwavelength Versus Broadband

An important discussion point raised in the literature concerns whether broadband systems are strictly necessary to assess mitochondrial function, particularly CCO, or whether discrete multiwavelength systems can offer equivalent insights. Reviews by Bale et al. and Leadley et al. have demonstrated that under certain conditions, systems using a carefully selected set of discrete wavelengths can provide reliable quantification of chromophores, including oxCCO.^22^^,^^23^ In simulation studies presented by Sudakou et al., it is shown that by optimally configuring around the CCO absorption band and accounting for detector responsivity, discrete multiwavelength systems can begin to approach the accuracies of broadband systems in CCO contrast, with reported uncertainties as low as 0.4 to 0.47  μmM.^59^

In a recent investigation by Caredda et al., a digital instrument simulator was created to identify the best wavelength selection across the visible and NIR spectrum, to resolve hemoglobin and cytochrome biomarkers.^60^ In each of the aforementioned publications, broadband NIRS is identified as the gold standard, offering superior chromophore separation and robustness against model errors and spectral overlap. Moreover, the commercial availability of some optimally placed wavelengths remains limited, posing a practical constraint to widespread deployment of such discrete systems.

### 4.2 Outlook on Future Designs

There are many new advancements in photonic components that could drive the future design considerations of bNIRS instruments, making them more effective in clinical monitoring, more robust to environmental changes, portable, miniature, and more economical.

The introduction of supercontinuum lasers and the continual advancements in laser instrumentation, with hyperspectral imaging already able to measure metabolism via changes in redox state of CCO,^61^ could form a replacement for the white-light sources used in bNIRS currently. White-light LEDs are used for skin-oxygenation measurements currently, with easily attainable devices that cover the visible spectrum and are more efficient than incandescent lamps. Phosphor converted LEDs (PC-LED) offer a spectrum that falls across the NIR region of light,^62^ making them candidates for a new fiber-coupled source or even a direct-to-skin device, reducing the amount of fibers used per system.

There is a similar shift in detector technologies. Although CCDs have historically been the dominant sensor type in bNIRS systems, we anticipate the field to experience a gradual transition toward CMOS-based detectors. CMOS sensors offer several advantages, including lower power consumption, reduced cost, and easier integration into compact devices. Typically, the CCDs are selected for their low noise floor and high sensitivities, in low-light applications; however, recent generations of CMOS sensors have demonstrated noise and sensitivity characteristics that approach or surpass traditional CCDs, particularly in the visible and near-infrared regions. A number of prevalent spectrometer and sensor manufacturers (e.g., Hamamatsu, Ocean Optics) have begun offering CMOS sensor-based detectors, which boast similar performance in NIRS spectral ranges, with significantly reduced form factors.

A review by Wang et al. investigated the published strategies for on-chip spectrometers, with indications of applications from research to commercial, industrial to civilian, and wearable to implantable.^63^ Calafiore et al. proposed a digital planar hologram (DPH) to replace the dispersive grating. By integrating multiple holograms on a single chip, they can increase spectral range and offset performance decrements from miniaturization. The validation device produced a spectral range of 148 nm and a resolution of 0.15 nm within a footprint of $2\;{\mathrm{cm}}^{2}$.^64^ However, there is yet to be seen a commercial implementation of this technique. We can also look to Fourier Transform (FT)-based spectroscopy, which is described quite early by Le Coarer et al.^65^ The implementation originally uses moving mirrors to create a tunable optical path length, which allows for measured intensities at various wavelengths. Kita et al. and Pohl et al. developed on-chip methods of FT spectroscopy that use industry-standard silicon photonics, with resolutions ≤2  nm and device footprints $∼1\;{\mathrm{cm}}^{2}$.^66^^,^^67^ A few techniques implement a wavelength filter directly onto the detector array, which reduces the size of instruments considerably. This has been done with polymer-based color filters,^68^ colloidal quantum dot (CQD) filters,^69^ and plasmonic metal filters.^70^ Reaching footprints as small as $2\;{\mathrm{cm}}^{2}$; these strategies, with the others mentioned, are in prime position to be implemented into personal, wearable devices, as they are now of comparable sizes to the sensors one can typically find in smartphone cameras.

During the editorial process for this review, Eskandari et al. reported in a preprint a combined broadband and time-domain NIRS system, enabling simultaneous acquisition of spectral and time-resolved information, namely, for the absolute quantification of oxidized and reduced CCO.^71^ Although this work appeared after the cutoff date for our review, it represents a promising direction in hybrid instrumentation.

**Fig. 8:**
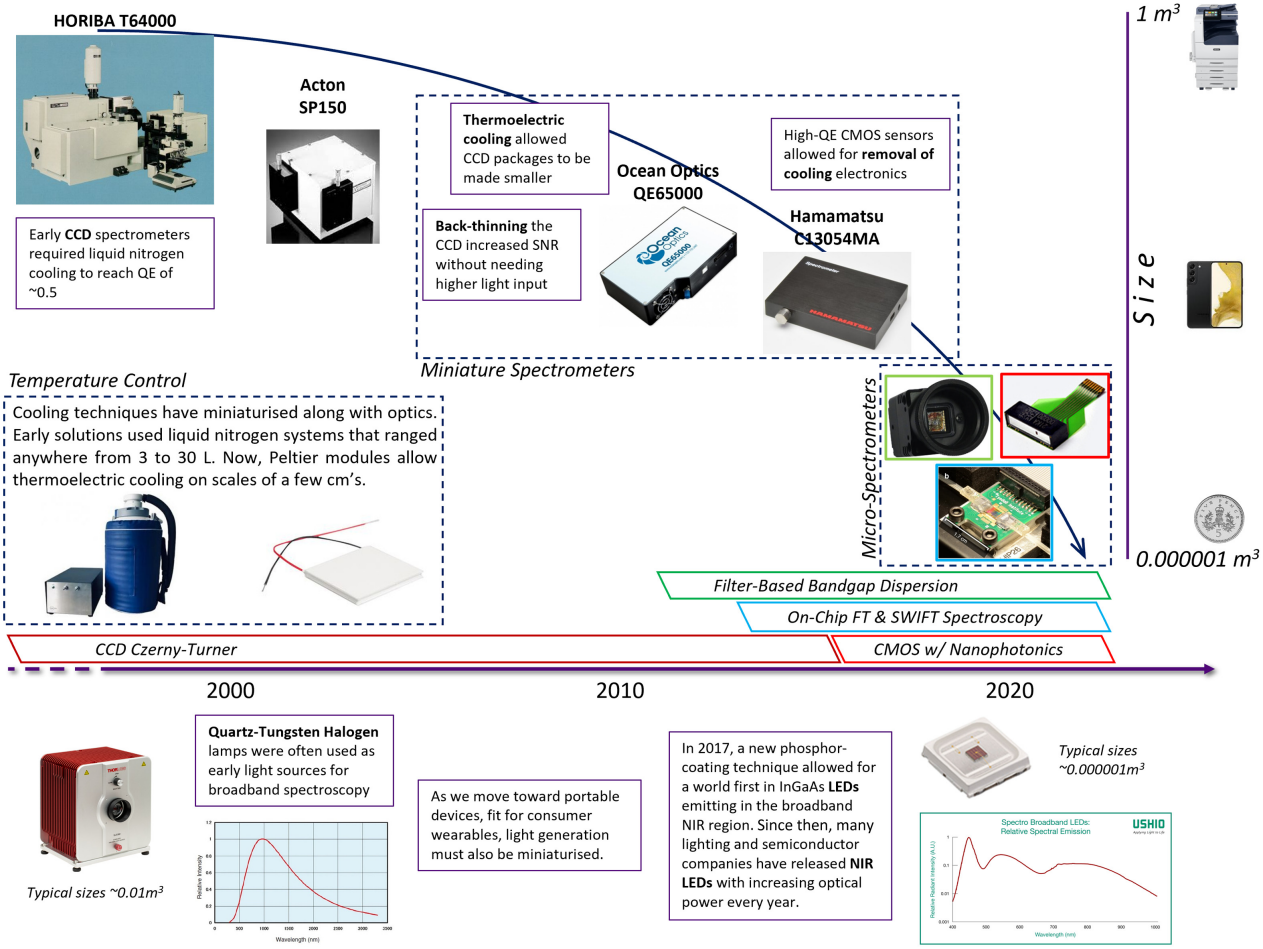
Timeline of major developments in the miniaturization of spectrometers, defining three generations of spectrometers, regular, miniature, and micro. Key developments, which made each generation accessible, are highlighted, including a broad view of the configurations explored in each generation. Developments have only moved away from traditional CZ CCD configurations in the last decade. Size comparison provided with everyday objects on the right.

An outline of the previous, current, and future generations of bNIRS system design can be seen in Fig. 8. Considerations have been made to the sources and detectors as they primarily drive the size of the system, and the innovations that have occurred to allow movement across each generation, i.e., changes in cooling devices, back-thinning CCDs, and alternate spectrometer configurations.

## 5 Conclusion

There are continued efforts to make more effective bNIRS systems each year, with sights set on making them more efficient, convenient, and commercially viable; addressing the urgent need for noninvasive measurements of tissue metabolism. It can be seen through the current system trends that there is a need for a greater number of channels, increasing information density with each scan, as well as a need for the systems to become smaller and more portable.

As portable technology around the globe advances, for mobile phones and personal healthcare devices, photonic components have become smaller and more efficient. CMOS sensors promise lower power consumption and better integration into small-scale devices, new spectrometer configurations allow for smaller form factors than ever before, and broadband LEDs remove the need for static fiber-coupled light sources. These could forecast new bNIRS systems with these micro components being as portable as current fNIRS devices while able to conduct the metabolism measurements of their full-scale clinical counterparts.

## Data Availability

The data used in this article are from the content and metadata of previous publications, available online. The article does not report data, and so, the data availability policy is not applicable to this article.
